# Cleavage of cell junction proteins as a host invasion strategy in leptospirosis

**DOI:** 10.1007/s00253-023-12945-y

**Published:** 2024-01-10

**Authors:** Preeti Kumari, Suhani Yadav, Sresha Sarkar, Padikara K. Satheeshkumar

**Affiliations:** Department of Botany, Institute of Science, Banaras Hindu University, Varanasi, Uttar Pradesh 221005 India

**Keywords:** *Leptospira*, Leptospirosis, Protease, Cell junction proteins, Pathogen invasion

## Abstract

**Abstract:**

Infection and invasion are the prerequisites for developing the disease symptoms in a host. While the probable mechanism of host invasion and pathogenesis is known in many pathogens, very little information is available on *Leptospira* invasion/pathogenesis. For causing systemic infection *Leptospira* must transmigrate across epithelial barriers, which is the most critical and challenging step. Extracellular and membrane-bound proteases play a crucial role in the invasion process. An extensive search for the proteins experimentally proven to be involved in the invasion process through cell junction cleavage in other pathogens has resulted in identifying 26 proteins. The similarity searches on the *Leptospira* genome for counterparts of these 26 pathogenesis-related proteins identified at least 12 probable coding sequences. The proteins were either extracellular or membrane-bound with a proteolytic domain to cleave the cell junction proteins. This review will emphasize our current understanding of the pathogenic aspects of host cell junction-pathogenic protein interactions involved in the invasion process. Further, potential candidate proteins with cell junction cleavage properties that may be exploited in the diagnostic/therapeutic aspects of leptospirosis will also be discussed.

**Key points:**

*• The review focussed on the cell junction cleavage proteins in bacterial pathogenesis*

*• Cell junction disruptors from Leptospira genome are identified using bioinformatics*

*• The review provides insights into the therapeutic/diagnostic interventions possible*

## Introduction

Leptospirosis is an infectious zoonotic disease caused by bacteria belonging to the genus *Leptospira*. These Gram-negative aerobic organisms are either free-living non-pathogenic forms or pathogenic forms. The pathogenic forms are grouped into 17 species and represent > 250 serovars (Picardeau 2017). Leptospirosis mainly occurs in tropical and subtropical areas where heavy rainfall and poor sanitation facilities are common. The disease is significantly underreported due to inept diagnostic methods and the symptoms match with many other bacterial and viral infections. At the global level, 1.03 million new cases of leptospirosis are reported annually with a mortality rate of more than 58,900 (Costa et al. 2015). Findings also suggest that patients with leptospirosis are prone to coinfection with many other pathogens and may pose a serious threat to the treatment options and well-being of these patients (Suppiah et al. 2017).

**Fig. 1:**
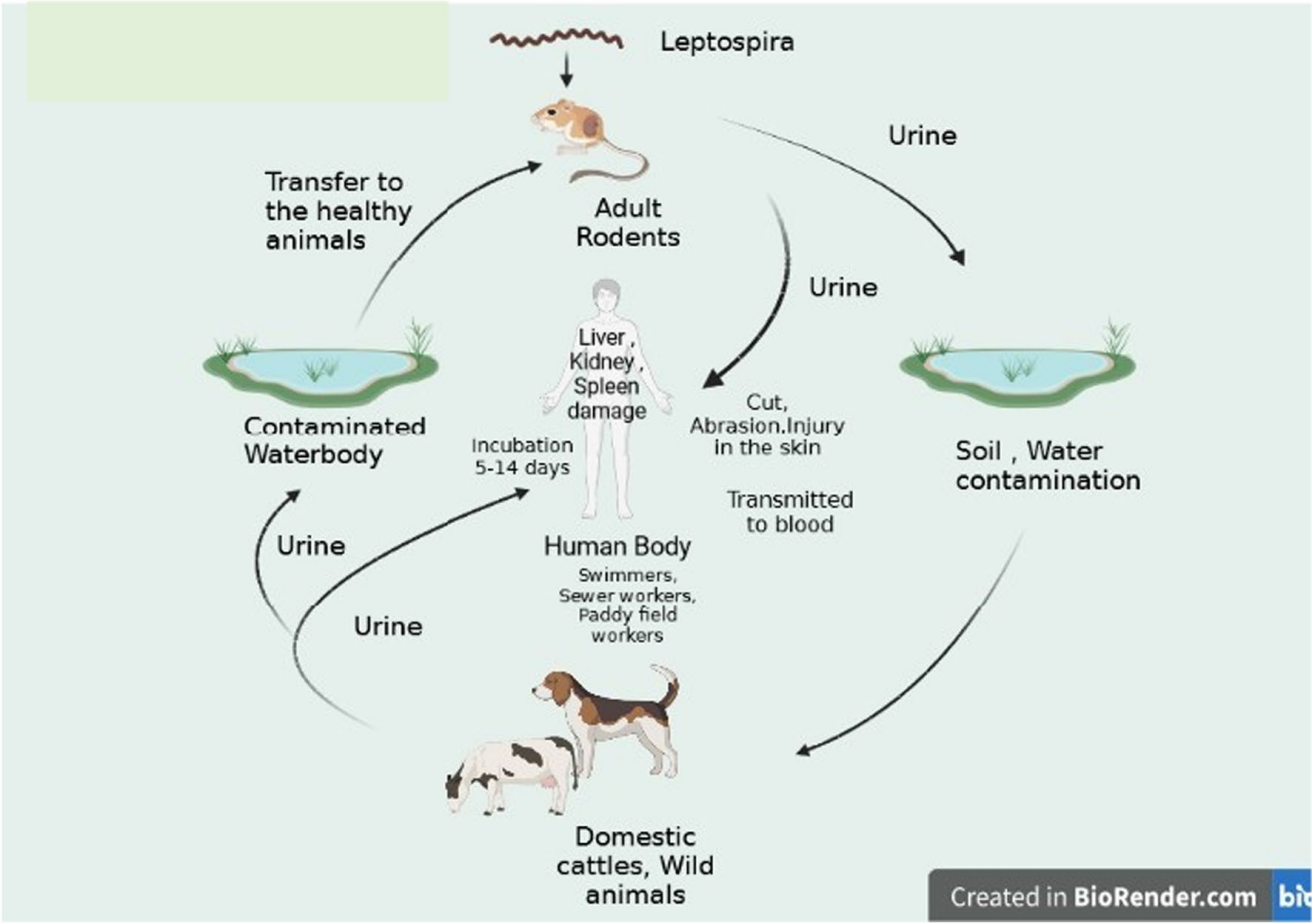
Transmission cycle of leptospirosis. The diagram shows the transmission dynamics in leptospirosis between rodents, wild and domestic animal reservoirs, and the environment. Pathogens infect humans by contact with an infected host or by contaminated water or soil. *Leptospira* invades through skin lesions and/or mucous membranes (Levet, 2015). This picture is created by BioRender.com

*Leptospira* infects a spectrum of both wild and domestic mammals, and once infected, these animals act as reservoir hosts, contaminating the environment, particularly water through their excreta. The pathogens may remain viable for days to weeks in soil and water with a neutral pH and are easily transmitted from infected soil or water to their host organisms (Russell et al. 2018). These spiral-shaped, highly motile organisms can cross through skin abrasions, conjunctiva, or intact mucous membranes (Wunder et al. 2016). Once the pathogen enters the body, it comes into the bloodstream by damaging the endothelial linings of blood vessels and disseminating all over the tissues and organs. Humans are infected with *Leptospira* through occupational exposure and living in rodent-infested, flood-prone urban slums. The transmission cycle can be seen in Fig. 1.

Chronic leptospirosis affects multiple organs including the liver, brain, eyes, kidneys, and lungs, causing jaundice, kidney failure, pulmonary hemorrhage, meningitis, uveitis, and conjunctivitis (Levett 2001). To enter the host body, the pathogen must cross epithelial and endothelial barriers. During the invasion, pathogenic leptospires adhere to the extracellular matrix (ECM) and degrade it. Pathogenic leptospires express extracellular proteases, most likely metalloproteases for the degradation of host proteins and proteoglycans while those were not produced by non-pathogenic strains (Da Silva et al.^2018^). Proteases released by the pathogenic strains during the initial phases of infection may play a crucial role in the invasion process and also in defending and averting the immune reaction of the host (Fraga et al. 2014).

To date, very few studies have been conducted experimentally to identify and characterize *Leptospira*l proteases (Dhandapani et al. 2018; Thoduvayil et al. 2020; Amamura et al. 2017; Kumar et al. 2022; Anu et al. 2018; Sato and Coburn 2017; Martinez-Lopez et al. 2010). To understand *Leptospira*l pathogenesis, it is mandatory to identify and characterize proteins mediating interactions with host components. As the whole-genome sequence data of many pathogenic and non-pathogenic strains of *Leptospira* is available, it is easy to compare these sequences with bio-informatics tools to predict proteins with a role in pathogenesis.

The review explores the pathogenesis mechanism, especially the cleavage of cell junction proteins as a critical step in the invasion process by *Leptospira*. While reports are plenty on many intracellular pathogens and their invasion mechanism, very little is known about *Leptospira*. Even though the role of many proteins in the ECM component interaction as part of the invasion process is known (reviewed by Daroz et al. 2021; Vieira et al. 2014; Fernandes et al. 2016), studies on the latter stage, which involves the cleavage of cell junction proteins to gain entry to the circulatory system are not available. In this review, along with the compilation of cell junction proteins and their functional aspects reported from *Leptospira*, a comprehensive genome analysis to identify the orthologs of the pathogenesis-related proteins reported from common intracellular bacterial pathogens was also performed. The computational analysis identified more than 10 pathogenic proteins based on the sequence similarity between the pathogenesis-related proteins and it will pave the way to study their role in invasion and pathogenesis in leptospirosis.

## Disrupters of epithelial junction during infection

Epithelial cells serve as the first barrier to prevent the entry of pathogens (excellently reviewed by Rogers et al. 2023; Backert et al. 2017; Zheng et al. 2021, and many more) such as *Pseudomonas aeruginosa* (Curran et al. 2018*)*, *Helicobacter pylori* (Chmiela and Kupcinskas 2019*),* Enteropathogenic *E. coli* (Singh and Aijaz 2015), *Clostridium difficile* (Czepiel et al. 2019), and *Clostridium perfringens* (McClane 2001) into the circulatory system and internal organs. The epithelial cell layers on one side form a barrier between internal organs and external invading pathogens but on the other side, it also serves as an infectious foothold for the pathogens as an entry port to disseminate into deeper tissues (Ashida et al. 2011). They not only serve as a physical barrier rather they also serve as a physiological barrier by secreting some chemicals such as lysozymes in saliva and tears, hydrochloric acid in the stomach, and many antimicrobial peptides (reviewed by Johnstone and Herzberg 2022; Brzoza et al. 2021; Kim et al. 2023; Wang et al. 2019) to prevent the entry of pathogens. Infection can happen when these barriers have been disrupted as in wounds and burns. In the absence of wounding and disruption, pathogens cross epithelial barriers by establishing a link through adhesion or colonization on these surfaces (Bonsor and Sundberg 2019; Ansari and Yamaoka 2019). The epithelial cell layers also serve as barriers to the free passage of foreign molecules (Zheng et al. 2021).

**Fig. 2:**
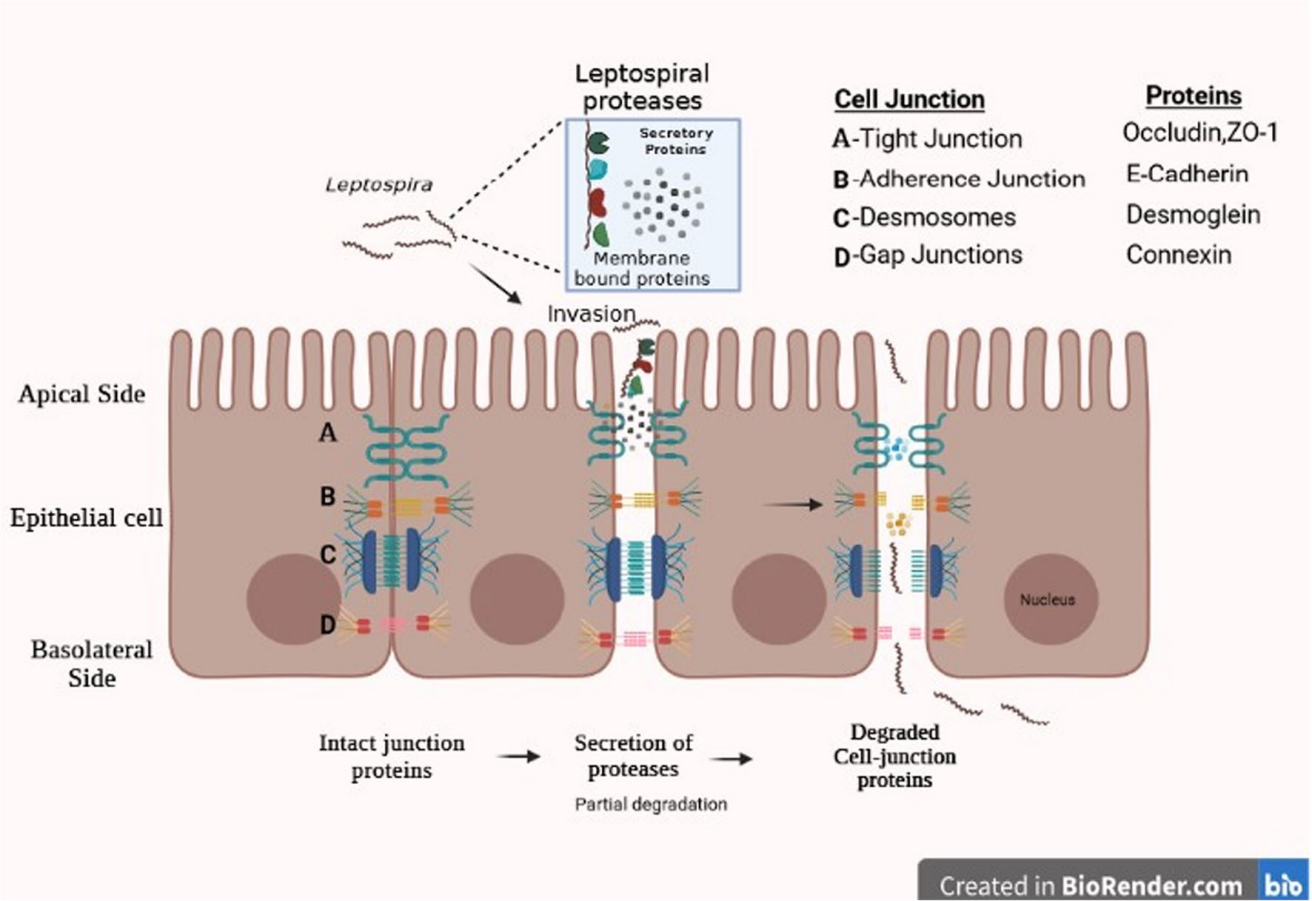
Schematic representation of the disruption of epithelial cell junctions by *Leptospira*. The barrier is composed of tight junctions, adherens junctions, desmosomes, and gap junctions. The infection affects the cell junction protein in two ways. Changes in the expression and location of these proteins (lead to changes in the epithelial barrier function) and the direct action of secreted and membrane-bound proteases disrupt the cell junction structures. Pathogens like *Leptospira*, using proteases target these cell junction proteins to cross themselves from the apical to basal side thus disrupting epithelial barrier function

The epithelium is a highly organized structure maintained by cell junctions. Cell junctions are complex multi-protein structures that provide contact among and between cells and ECM in animals. Thus, cell junctions help in holding animal cells together, maintain the paracellular barrier of epithelial cells, and control paracellular permeability (Garcia et al. 2018). There are mainly three types of cell junctions: adherens or anchoring junctions, tight or occluding junctions, and gap or communicating junctions. Different types of proteins are involved to form cell junctions such as cadherins, integrins, connexions, occludins, and claudins. Epithelial cell junctions show selective permeability and thus maintain polarity across the epithelium (Horowitz et al. 2023; Adil et al. 2021). Disruption of this barrier leads to the paracellular movement of molecules along with bacteria, viruses, toxins, etc. into the systemic circulation. Bacterial pathogens produce proteins to disrupt epithelial cell junctions by targeting these junctional proteins to get access to blood circulation (Al-Obaidi and Desa 2018; Zheng et al. 2021). A general representation of cell junction disruption by the bacterium is shown in Fig. 2.

The pathogens developed various mechanisms to circumvent the epithelial cell barrier by expressing several kinds of virulence factors, toxins, proteases, etc. during the course of invasion. Enteric Pathogens like enterohaemorrhagic *E. coli*, Shigella species, and enteropathogenic Yersinia employ the secretion systems type 3, 4 and 5 (type 3/4/5 secretion system—T3SS/T4SS/T5SS) to inject toxic proteins into the host cells, leading to the disarray of the host cell cytoskeleton, facilitating the invasion of the pathogen (reviewed by Whelan et al.^2020^; Viana et al. 2021). *Pseudomonas aeruginosa* uses a biofilm-like matrix for the transmigration process and uses multiple approaches to gain entry into the host cells, such as T2SS, quorum sensing, T3SS, and chemicals like N-(3-oxododecanoyl) L-homoserine lactone. The combined use of toxin and protease hamper the cell junction integrity allowing pathogen entry into the host cells (reviewed by Golovkine et al.^2018^; Qin et al.^2022^; Pont et al.^2022^). To cross the host blood barrier, *Neisseria meningitides* disrupt the endothelial permeability and it was proposed that *N. meningitides* recruit proteins involved in the formation and stabilization of adherens and tight junction into the cortical plaques, which is a molecular complex formed under the bacterial colonies, leading to the opening of intercellular cell junction (Coureuil et al. 2012). *Helicobacter pylori* use a complex virulence mechanism that supports the attachment, colonization, evasion, and modulation of the host immune system, activation of many virulence pathways, and disruption of the cell junctions to gain entry into the host cells (Baj et al. 2020). Downregulation of the expression of cell junction proteins is one of the effects of intracellular pathogen infection. Spontaneous bacterial peritonitis (SBP) is a severe condition of liver cirrhosis caused by *E. coli* and *Proteus mirabilis* (*P. mirabilis*). Haderer and co-workers found that the mucus layer of the intestine was thin in patients suffering from SBP. It is because, in SBP, E-cadherin and occludin proteins are downregulated in adherens and tight junctions respectively and for this reduction, bacterial-host direct interaction is required (Haderer et al. 2022).

Extracellular and membrane-bound proteases of pathogenic bacteria play a crucial role in the invasion process (Linz et al. 2023; Singh and Phukan 2019). One of the widely studied serine proteases, HtrA are expressed by several pathogenic bacteria such as *C. jejuni*, *Salmonella enterica*, EPEC, *Proteus mirabilis*, and *Yersinia enterocolitica* target E-cadherin during infection (Hoy et al. 2010; Backert et al. 2018; Song et al. 2021). Almost every bacterium causing infectious disease expresses at least one homolog of the HtrA family (Rawlings et al. 2008). In the case of *E. coli,* DegP, DegQ, and DegS show structural similarity with HtrA proteins of other Gram-negative bacteria (Waller and Sauer 1996). Other than E-cadherins, tight junction proteins such as claudins also act as a target for HtrA in *C. jejuni* (Sharafutdinov et al. 2020). In the animal models, knocking out of E-cadherin from the host or deletion of the HtrA gene from the pathogen prevented the pathogen’s entry into the host (Cao et al. 2021) evidencing that HtrA or HtrA homologs alone can control the pathogenesis in intracellular pathogens. A trypsin-like serine protease domain containing Ssp1 protease from *Aeromonas hydrophila* is responsible for the downregulation of a tight junction protein occludin (Feng et al.^2022^). InlA secreted by *Listeria monocytogenes* (Nikitas et al. 2011) is used by the pathogen to cross the intestinal barrier by interacting with E-cadherin. Some pathogens also activate the host’s protease that disrupts the epithelial barrier as in the case of periodontitis. In periodontitis, neutrophils of the host’s immune system get activated and these neutrophils start producing neutrophil elastase (NE) which further damages the E-cadherin, occludins, and desmoglein-1 of the oral epithelial tissue (Hiyoshi et al. 2022). *Pseudomonas aeruginosa* and *Serratia marcescens* secrete toxins ExlA (Exolysin) and ShlA (*Serratia* hemolysin A) respectively. These pore-forming toxins bind with host cell receptors and cause an increase in cytosolic Ca^2+^ that further triggers a host cell transmembrane metalloprotease ADAM10 activation leading to E-cadherin and VE-cadherin cleavage (Reboud et al. 2017). Bacterial proteases target cell junction proteins for the adhesion and invasion process are listed in Table 1.

**Table 1:** Overview of bacterial proteins targeting cell junction proteins for the invasion process and their important features

Protein	Source	Group	Location	Cofactor	Domains	Function	Receptor	Reference
FadA	*Fusobacterium nucleatum*	Kinase	Membrane/secreted	-	Riboflavin Kinase	Phosphorylation	E-cadherin	Rubinstein et al. (2013)
InlA and InlB	*Listeria monocytogenes*	Leucine-rich repeat	Membrane/secreted	-	Leucine Rich Repeat	Heparin-binding	E-cadherin	Ortega et al. (2017)
PsaA	*Streptococcus pneumoniae*	Metalloprotease	Surface protein	Mn^2+^, Zn^2+^	Prokaryotic membrane lipoprotein lipid attachment site profile	Cell adhesion	E-cadherin	Anderton et al. (2007)
HtrA	*Helicobacter pylori, Escherichia coli, Shigella flexneri*	Serine protease	Periplasmic protein	-	PDZ	Proteolysis and stress response	E-cadherin	Tegtmeyer et al. (2017), Hoy et al. (2012)
SpeB	*Group A Streptococcus*	Cysteine protease	Secreted protein	-	C-terminal active site loop	Proteolysis, evasion of host immune response	Occludin and E-cadherin	Sumitomo et al. (2003)
LasB	*Pseudomonas aeruginosa*	Metalloprotease	Secreted	Ca^2+^, Zn^2+^	Neutral zinc metallopeptidases	Proteolysis	VE-cadherin	Golovkine et al. (2014)
BoNTHA	*Clostridium botulinum*	Metalloprotease	Secreted	Zn^2+^	Zn protease	Proteolysis	E-cadherin	Sugawara et al. (2010)
Als3/Sap5p	*Candida albicans*	Aspartate protease	Membrane/secreted	-	Agglutinin-like protein	Cell adhesion, virulence	E-cadherin	Phan et al. (2007)*,* Villar et al.(2007)
BFT	*Bacteroides fragilis*	Metalloprotease	Secreted	Zn^2+^	Prokaryotic membrane lipoprotein lipid attachment site profile	Proteolysis	E-cadherin,	Wu et al. (1998), Wu et al.(2007)
HRgpA, RgpB, Kgp	*Porphyromonas gingivalis*	Cysteine Protease	Secreted	Ca^2+^	Caspase-like domain	Proteolysis	E-cadherin	Katz et al. (2000)
GelE	*Enterococcus faecalis*	Neutral metalloprotease	Secreted	Zn^2+^, Ca^2+^	Neutral zinc metallopeptidases	Proteolysis	E-cadherin	Steck, et al. (2011)
Hla	*Staphylococcus aureus*	Alpha-hemolysin	Secreted	Ca^2+^	Aerolysin-like signature	Cytolysis, hemolysis, virulence	E-cadherin	Inoshima et al.(2011)
Delta toxin	*Clostridium perfringens*	Metal-binding protein	Secreted	Zn^2+^	Leukocidin/hemolysin toxin domain-containing protein	Cytolysis	E-cadherin	Seike et al. (2018)
Aerolysin	*Aeromonas hydrophila*	Alpha-hemolysin	Secreted	-	Aerolysin type toxins signature	Cytolysis/pore-forming	Occludin	Bucker et al. 2011
TcdA and TcdB	*Clostridium difficile*	Metalloprotease	Secreted	Zn^2+^, Mn^2+^, Mg^2+^	receptor-binding (CROPS) domain	Proteolysis	Occludin, ZO-1, ZO-2	Nusrat et al. 2001
UreB	*Helicobacter pylori*	Metalloprotease	Cytoplasmic	Ni^2+^	Urease	Virulence	Occludins	Wroblewski et al.^2009^
LAP	*Listeria monocytogenes*	Adhesin	Membrane	-	Cadherin domain signature	Cell adhesion	Claudin-1, occludin, and E-cadherin, ZO-1	Burkholder and Bhunia (2010)
HA/P	*Vibrio cholerae*	Metalloprotease	Secreted	Zn^2+^	Fungalysin/thermolysin propeptide (FTP) domain and a PepSY domain	Proteolysis	Occludin and ZO-1	Wu et al. 2000
ZOT	*Campylobacter concisus*	Hydrolase	Membrane-bound	-	Zona occludens toxin N-terminal	Virulence	ZO-1	Deshpande et al. 2016
VacA and CagA	*Helicobacter pylori*	Transporter	Membrane/secreted	-	Auto-transporter	Virulence	ZO-1	Krueger et al.^2007^

*FadA* *Fusobacterium* adhesion, *InlA and InlB* Internalin A and Internalin B, *PsaA* pneumococcal surface adhesin A, *HtrA* high temperature requirement A, *SpeB* streptococcal pyrogenic exotoxin B, *lasB* elastase B, *BoNTHA* botulinum neurotoxin hemagglutinin, *ALS3* agglutinin-like sequence 3, *Sap5p* secreted aspartic proteases 5 pep, *BFT* Bacteroides fragilis toxin, *HRgpA* hemagglutinin arginine gingipain A, *RgpB* arginine gingipain B, *Kgp* lysine gingipain, *GelE* gelatinase, *Hla* alpha hemolysin, *TcdA and TcdB Clostridium difficile* toxin A and B, *UreB* urease subunit beta, *LAP Listeria* adhesion protein, *HA/P* haemagglutinin/protease, *ZOT* Zona occludin toxin, *VacA* vacuolating cytotoxin autotransporter, *CagA* cytotoxin-associated gene A, *ZO* zonula occludens

## *Leptospira* and cell junction proteins

Cell junction proteins act as targets for proteases expressed by pathogenic *Leptospira* for their attachment and invasion. It was reported that the pathogenic form of *L. interrogans* infected cells loosens its adherens junction proteins, VE-cadherin (vasculo-endothelial-cadherin), p120-, alpha and beta-catenins, and tight junction proteins, actin, and ZO-1 from the original site at intercellular junctions (Sato and Coburn 2017). De Brito and co-workers observed a loss of expression of E-cadherin protein on the membrane of hepatocytes in the case of human leptospirosis. Also, the expression of E-cadherin in liver cells was absent in areas of the lobule; thus, a stable intercellular adhesion was missing (De Brito et al. 2006). According to the study of Martinez-Lopez and co-workers, the binding changes membrane permeability and allowed the free passage of molecules and the pathogen itself across the endothelial cell layers. Pathogenic strains dislocate the endothelial cell layers by targeting cell junction proteins and creating gaps in between to increase vascular permeability leading to swelling in lung alveoli and hemorrhage (Martinez-Lopez et al. 2010). Evangelista et al. (2014a, b) found that pathogenic *Leptospira* binds with VE-cadherin of endothelial cells through adhesin proteins and lipoproteins.

The tripeptide RGD (Arg-Gly-Asp) motif present on many proteins binds to integrins and is the most common peptide motif responsible for cell adhesion to the ECM (Makowski et al. 2021). RGD motif is present in several other pathogenic microorganisms like *Helicobacter pylori* (Bub et al.^2019^), *B. pertussis* (Leininger et al.^1991^), and *Mycobacterium tuberculosis* (Dubey et al.^2021^). Cavenague and co-workers characterized an RGD motif-containing protein LIC12254 expressed by pathogenic species of *Leptospira* but not by intermediate or saprotrophic species through in silico analysis. They showed that recombinant LIC12254 interacts with human αVβ8 integrin and the α8 integrin chain via the RGD motif, while in the recombinant protein lacking RGD motif, binding was abolished (Cavenague et al. 2023). These results suggest that LIC12254 is an outer membrane protein that shows adhesion with human integrins via the RGD domain and has a role in leptospirosis. Recombinant LIC10831 (LRR containing protein) and recombinant dermal human microvascular endothelial cell line (HMEC-1) were generated by Eshghi et al. (2019) and using techniques like SPR (surface plasmon resonance) and ELISA (enzyme-linked immunosorbent assay); it was shown that rLIC10831 bind with endothelial cells. The binding was enhanced by Zn^2+^.

Kochi and co-workers cloned, expressed, and purified two novel putative surface-exposed hypothetical lipoproteins LIC11711 and LIC12587. Both proteins are conserved among pathogenic strains of *Leptospira interrogans*. Both recombinant proteins show binding affinity to E-cadherin and laminin, so provide initial adhesion to host epithelial cells and both interact with E-cadherin in a dose-dependent manner (Kochi et al. 2019). Pinne et al. (2010) identified OmpL37 (LIC12263) to determine the binding affinity of protein to host tissue by ELISA. OmpL37 is shown to bind with aortic as well as human skin elastin protein. It also binds with other ECM proteins like laminin, fibrinogen, and fibronectin. The binding of human skin elastin to recombinant OmpL37 as well as *Leptospira interrogans* indicates that OmpL37 helps pathogenic *Leptospira* to bind with host tissues via elastin. Moreover, it has been shown that OmpL37 is present only in pathogenic sp. of *Leptospira* but not in saprotrophic ones. Pereira et al. (2017) identified two surface protein-encoding genes Lsa25.6 (LIC13059) and Lsa16 (LIC10879), cloned them, and expressed them in the *E. coli* system and reported that both the recombinant proteins were adhesins, interacting with laminin in a dose-dependent manner. But when it comes to binding with epithelial cells, only Lsa16 shows binding with E-cadherin. Leptospira genome contain many genes showing sequence similarity with pathogenic proteases (Table 2).

**Table 2:** Bacterial protease targeting cell junction showing sequence similarity in the genome of different *Leptospira* species (for the gene names please refer to Table 1)

Gene	Source organism	Binding with	Homologous with gene	Gene ID (NCBI/Uniprot)	Present in *Leptospira* species
*PsaA*	*Streptococcus pnemoniae*	E-cadherin	TroA-like (manganese ABC transporter substrate-binding protein)	WP_193819214.1	*Leptospira borgpetersenii*
*HtrA*	*Helicobacter pylori*	E-cadherin, occludin, claudin	periplasmic serine protease	WP_010679781.1	*Leptospira interogans*
Invasin	*Yersinia pseudotuberculosis*	Integrin	Immunoglobulin-like protein A	C8CS17	*Leptospira interrogans*
Internalin	*Listeria monocytogenes*	E-cadherin	FVE87_07955 (Internalin)	A0A6G9EJ20	*Leptospira interrogans*
*FadA*	*Fusobacterium nucleatum*	E-cadherin	fadA	A0A2H1XHX8	*Leptospira interrogans*
*LasB (Elastase)*	*Pseudomonas aeruginosa*	E-cadherin	GluZincin (M4 family metallopeptidase)	WP_194490215.1	*Leptospira borgpetersenii*
			Peptidase M 4_C (M4 family metallopeptidase)	WP_000319896.1	*Leptospira interrogans*
*BFT*	*Bacteroides fragilis*	E-cadherin	ZnMc (matrix domain protein)	TGK01715.1	*Leptospira semungkisensis*
*HtrA*	*Campylobacter jejuni*	Occludins and claudins	Trypsin-like peptidase domain-containing protein	MCC5814724.1	*Leptospira* sp.
*ureB*	*Helicobacter pylori*	Occludins	Urease subunit alpha	MBE8362490.1	*Leptospira borgpetersenii*
*LAP*	*Listeria monocytogenes*	Claudins-1, occludin, and E-cadherin, ZO-1	Internalin_H (InlB B-repeat-containing protein)	WP_193823337.1	*Leptospira borgpetersenii*
*HA/P*	*Vibrio cholerae*	Occludin and ZO-1	GluZincin (M4 family metallopeptidase)	WP_194490215.1	*Leptospira borgpetersenii*
			HA/P1	Q79B72	*Leptospira interrogans*
*CagA*	*Helicobacter pylori*	ZO-1	cagA	Q72PV3	*Leptospira interrogans*

## Cell junction disrupter orthologs in *Leptospira* genome

During the last decade, research work targeting the pathogenesis mechanism of leptospirosis has seen an upsurge in the identification of components involved in the adhesion, colonization, immune evasion, and establishment of pathogens in the host system. Even though the complete genome is available for many pathogenic and non-pathogenic strains (Ramli et al. 2021; Vincent et al. 2019; Thibeaux et al. 2018) and many studies comparing the genomes are published, a clear picture of the pathogenesis mechanism is not available. In *Leptospira*, as per the current data, nearly 10 proteins were found to act on the cell junction proteins of the host system and play a role in the invasion/colonization process (Evangelista et al. 2014a, b; Eshghi et al. 2019; Pinne et al. 2010 and many more). Many of these reports were established using the recombinant proteins (Table 3), and few of them were using the protein purified from the culture medium.

**Table 3:** Pathogenic proteins expressed in various heterologous systems (for the gene names please refer to Table 1)

Gene	Source	Heterologous system	Biological activity	Reference
FadA	*Fusobacterium nucleatum*	*E. coli* BL21(DE3)	Adhesion to vascular endothelial (VE)-cadherin	Xu et al. 2007
HtrA	*Helicobacter pylori*	*E. coli* BL21(DE3)	Cleaves E-cadherin to disrupt intercellular adhesion	Tegtmeyer et al. 2017
PsaA	*Streptococcus pneumoniae*	*S. aureus*	Interact with nasopharyngeal epithelial cells	Hu et al. 2021
SpeB	Group A *Streptococcus*	*E. coli* rosetta	Shows proteolytic activity against human occludin and E-cadherin	Deng et al. 2022
BoNTHA	*Clostridium botulinum*	*E. coli* rosetta	Binds to E-cadherin and inhibits E-cadherin-mediated cell-cell adhesion	Amatsu et al.^2023^
Als3/Sap5p	*Candida albicans*	*Escherichia coli* strain XL-1 Blue	Invades and damages epithelial cells via Als3-E-cadherin interactions	Laforce-Nesbitt et al. 2008
GelE	*Enterococcus faecalis*	*E. coli* BL21 (DE3)	E-cadherin degradation	Kazemian et al. 2019
Delta toxin	*Clostridium perfringens*	*E. coli* BL21 (DE3)	Reduces the cellular levels of adherence junction protein E-cadherin via increasing the level of ADAM10	Manich et al. 2008
Aerolysin	*Aeromonas hydrophila*	BL21(DE3)/pLysS	Impairs epithelial integrity by promoting TJ protein redistribution	Diep et al. 1999
tcdA and tcdB	*Clostridium difficile*	*B. megaterium*	Dissociates occludin, ZO-1, and ZO-2	Yang et al. 2008
*ureB*	*Helicobacter pylori*	E. coli BL21(DE3)	Involves in occludin internalization and barrier dysfunction in gastric epithelial cells	Mao and Yan 2004
LAP	*Listeria monocytogenes*	*Lactobacillus paracasei*	Opens epithelial barrier via cellular redistribution of the epithelial junctional proteins claudin-1, occludin, and E-cadherin	Koo et al. 2012
ZOT	*Campylobacter concisus*	*E. coli* BL21 (DE3) pLacI	Damages intestinal epithelial barrier	Mahendran et al. 2016
Mce (LIC11859)	*Leptospira interrogans* serovar Copenhageni M-20	*E. coli* BL21 (DE3)	Binds with ECM, plasma components, and beta 2 integrins	Cosate et al. 2016
LIC13059 and	*L. interrogans* serovar Copenhageni	E. coli BL21 (DE3)	Interacts with E-cadherin	Pereira et al. 2017
LIC10879				
LipL21 and	*L. interrogans* serovar Copenhageni strain M20	*E. coli* BL21 (DE3)	Interacts with a variety of endothelial and epithelial cell lines	Takahashi et al. 2021
LipL41				
LIC11711 and	*L. interrogans* serovar Copenhageni M20	*E. coli* BL21 (DE3)	Binds with E-cadherin and laminin	Kochi et al. 2019
LIC12587				
OmpL1 (LIC11574)	*L. interrogans* sv. Copenhageni st. Fiocruz F1–130	*E. coli* expression strain KS330	Binding of pathogenic *Leptospira* to cadherin, damages the vascular system	Evangelista et al. 2014a, b
*LIC12254*	*L. interrogans* serovar Copenhageni	*E. coli* BL21 Star (DE3)	interacts with human αVβ8 integrin and the and α8 integrin	Cavenague et al. 2023
LIC10831	*L. interrogans* sv. Copenhageni strain Fiocruz L1-130	*L. interrogans* serovar Manilae strain L495	Binds to E- cadherin and VE-cadherin	Eshghi et al.^2019^
LIC10091 (LipL40)	*L. interrogans* serovar Copenhageni strain Fiocruz L1-130	*E. coli* BL21 Star (DE3)	Shows adhesion to the human aorta, and skin elastin protein	Pinne et al. 2010

**Fig. 3:**
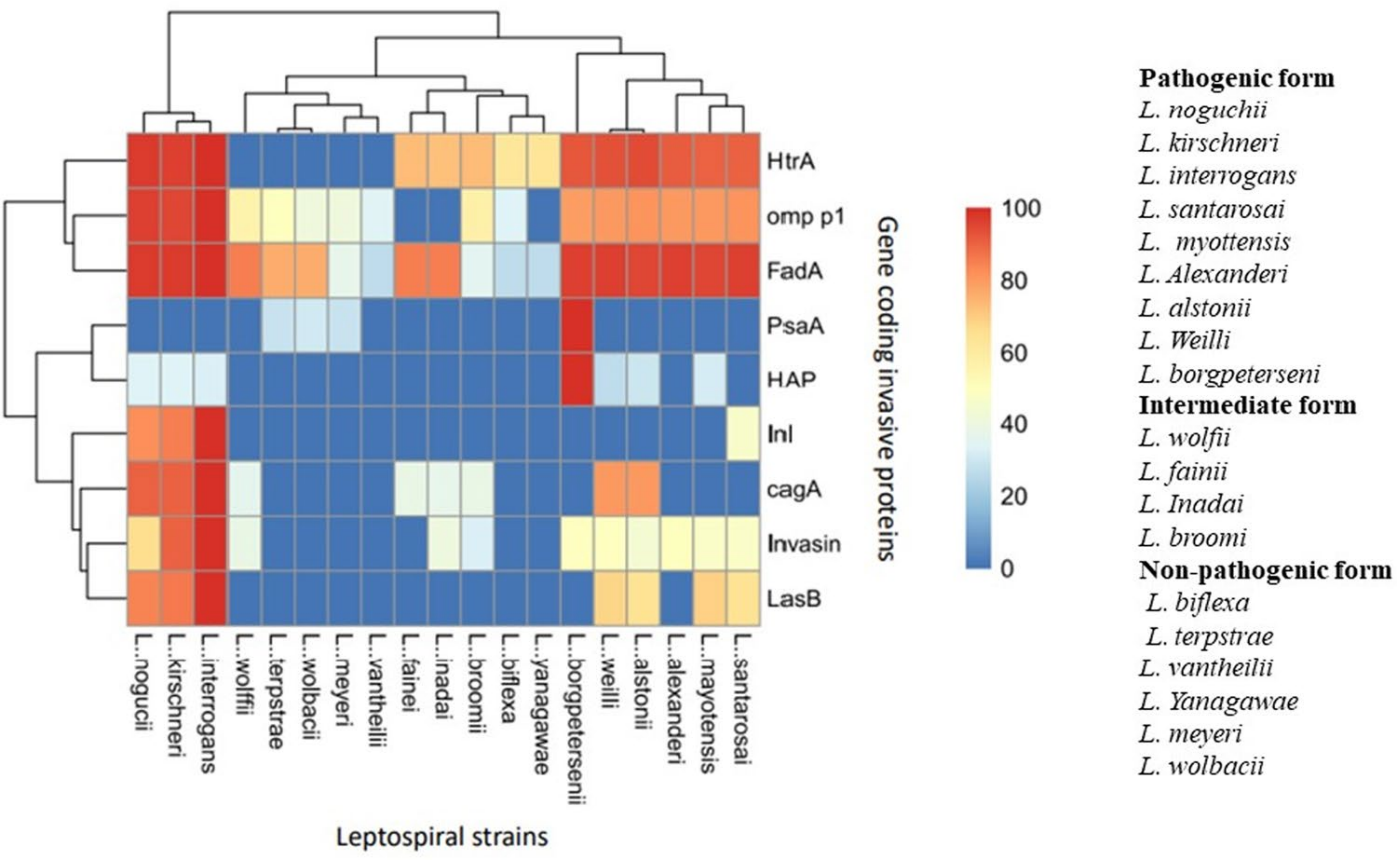
Comparative amino acid sequence analysis of nine pathogenic proteins in different species of *Leptospira*. Nine proteins involved in the invasion/pathogenesis caused by different human pathogens were used to search for similar sequences in *Leptospira*. The heat map compares the percent identity of a particular protein sequence from 19 species of *Leptospira* (9 pathogenic, 4 intermediate, and 6 saprophytic). Sequence similarity is shown by red (80% and above), yellow with 40–60% similarity, and shades of blue from 30% to no similarity. Pathogenic species show more expression of proteins involved in pathogenesis over intermediate or non-pathogenic forms of *Leptospira*. Species of pathogenic, intermediate, and non-pathogenic forms of *Leptospira* are mentioned on the right side of the figure

The similarities that exist in the invasion and pathogenesis machinery among different pathogenic, intracellular bacteria prompted us to look for the presence of some of the most widely reported and critical components of the pathogenesis machinery in the *Leptospira* genome. The pathogen-related gene sequences mainly involved in cell junction cleavage obtained from other pathogens were used as bait to look for similar sequences in the *Leptospira* genome. In some cases, instead of nucleotide sequence, the amino acid sequence was used for the search due to very low similarity results with the nucleotides. To explore more about the pathogenicity-related genes in the genome of *Leptospira*, seven proteins, proven experimentally to be involved in the invasion process of different intracellular pathogens were selected. The sequences collected from different strains of *Leptospira* including the pathogenic, non-pathogenic, and intermediate forms were used to check for the presence of domains making them active proteases/peptidases. Localization onto the outer membrane or to the secretome was another criterion for the selection of sequences. Depending on the number of domains and sequence similarities the sequences were grouped into three major clades delimiting the strains as per their pathogenicity. Pathogenic strains showed the presence of pathogenic proteins reported from other species (more than 80% similarity indicated by red-colored blocks in the heatmap). Among the strains, *L. interrogans* showed the presence of 7 out of 9 proteins in the genome with high sequence similarity (Fig. 3). HtrA and FadA were present in the intermediate forms indicating that these two pathogenic proteins may have a widespread distribution among the genomes of pathogenic and intermediate forms of *Leptospira*. Two proteins PsaA and HAP were present only in the pathogenic strain *L. borgpeterseni*. The genome of two non-pathogenic strains (*L. vantheilii* and *L. meyeri*) selected for the study lacks any of the pathogenesis-related protein sequences used in the study.

## Potential inhibitors of cell junction disrupters

Seven types of proteases are available on the MEROPS database, i.e., serine-, threonine, glutamate-, aspartate-, asparagine-, cysteine- and metalloprotease (Rawlings et al. 2018) which is the most common protease expressed by pathogenic bacteria. Protease inhibitors play a major role in the containment of many bacterial/viral diseases. So, it is a worthwhile practice to look for inhibitors and their applications in therapeutics. Out of 26 selected proteins from different pathogenic bacteria involved in invasion, nine were metalloproteases and five out of 12 selected proteases from *Leptospira* were also metalloproteases. The inhibitors of metalloproteases are mainly chenodeoxycholic acid, phosphinic acid-based pseudopeptide inhibitor, Raxibacumab, and phosphonamidate dipeptides (Sundar et al. 2023). The Zn^2+^ metalloprotease involved in the invasion/pathogenesis is represented by PsaA, LasB, BFT*,* and HA/P and can be inhibited by chenodeoxycholic acid, dithiothreitol, dithioerythreitol, and phosphinic acid-based pseudo peptides (Yang et al. 2011; Metz et al. 2019; Migone et al. 2009). UreB is a Ni^2+^-dependent protease found in many pathogenic bacteria. There are many natural and synthetic inhibitors of UreB reported in the literature (Loharch and Berlicki 2022).

HtrA is a serine protease expressed by a wide variety of pathogenic bacteria and contributes to pathogenesis directly (Zarzecka et al. 2019). It not only enables pathogens to survive in stressful conditions but also cleaves multiple host proteins such as E-cadherin and other extracellular matrix proteins (Wessler et al. 2017). Some of the HtrA inhibitors like camostat, gabexate, nafamostat mesylates (Amrutha et al. 2023), ecimicin (Choules et al. 2019), and rufomycin (Gao et al. 2015) have been developed against different pathogenic bacteria but show a harmful effect on human health. Hwang and co-workers (2021) designed and synthesized a peptide-based inhibitor JO146 using nanotechnology, which was not toxic to humans as well as other model organisms and effective only against *Chlamydia* (Hwang et al. 2021) and *H. pylori* (Hwang et al. 2022), but not effective against other pathogens like *Staphylococcus* sp., *Pseudomonas* sp., and pathogenic *E. coli*. Exploring the invasion mechanism in Leptospirosis further opens up new avenues for the identification and implementation of proteases against the disease.

## Conclusion

Leptospirosis shows an increased occurrence worldwide in the last few decades, mainly due to changes in climatic conditions. This made the environment more conducive for the survival and multiplication of reservoir hosts and the zoonotic epidemics will be a serious threat to the healthcare system of developing countries in the coming years (Limaye 2021; Prillaman 2022). Unlike many intracellular pathogens, which makes their presence ubiquitous, Leptospires are common in tropical and subtropical regions, affecting mainly the population of developing countries. The complex interaction predicted with climate change and disease occurrence by several studies indicates the necessity of having a thorough understanding of zoonosis to prevent it efficiently. Mining the genome and proteome data to identify novel genes and proteins which play crucial roles in pathogenesis is important in this process. Even though in the last few years, there were many publications on *Leptospira*l protein interaction with ECM, epithelial cell junction, and the immune components of the host, many questions are still unanswered. The major queries about the components involved in the attachment, invasion, and colonization process are only partly answered. Further, a mechanism of transmigration to different organs and circulatory system needs to be identified. There were very few studies on the biological characterization of pathogenic proteins in the model pathogenic strains of *Leptospira* making it difficult to conclude anything with the information available at present. The new orthologous reported in this review may help us to fill some gaps.
